# The impact of exposure to green or bluespace on dietary intake and food choices among adults—A systematic literature review

**DOI:** 10.1002/fsn3.4447

**Published:** 2024-11-04

**Authors:** Claire A. Gilbourne, Alan Scarry, Audrey C. Tierney, Eibhlís M. O’Connor

**Affiliations:** ^1^ School of Medicine University of Limerick Limerick Ireland; ^2^ Department of Biological Sciences University of Limerick Limerick Ireland; ^3^ Health Implementation Science and Technology Health Research Institute, University of Limerick Limerick Ireland; ^4^ Faculty of Education and Health Sciences, School of Allied Health University of Limerick Limerick Ireland; ^5^ Department of Nutrition, Dietetics and Sport La Trobe University Melbourne Victoria USA; ^6^ Health Research Institute, University of Limerick Limerick Ireland; ^7^ Alimentary Pharmabiotic Centre (APC), Microbiome Ireland University College Cork Cork Ireland

**Keywords:** bluespace, diet, food choices, greenspace, nutrition

## Abstract

Extensive research has shown that spending time in natural greenspaces has a positive impact on health. However, there is limited evidence regarding potential factors that may influence these effects. This review aimed to assess the strength of the evidence and potential impact of exposure to green and bluespaces on dietary outcomes in adults. Inclusion criteria for the review were based on the PICO criteria. Following PRISMA guidelines, an initial search of five databases was conducted: CINAHL, GreenFILE, AMED, Medline, and PubMed, accessed on 14th June 2021 and augmented by an updated rerun in January 2024. All studies used the Joanna Briggs Institute (JBI) Critical Appraisal Checklist for Analytical Cross‐Sectional Studies for quality assessment. Due to heterogeneity, a narrative synthesis was conducted to evaluate the relationships between the included studies. Four observational studies that reported diet‐related outcomes were included in the review, and participants within the studies ranged from 554 to >350,000 participants. Other health outcomes, including physical activity and obesity, were also reported. Two studies found that dietary patterns were not correlated with exposure to greenspace. Due to the small number of articles retrieved and the paucity of evidence, the findings must be interpreted cautiously. In conclusion, further research is essential to clarify the intricate mechanisms involved in greenspace‐related health benefits. Additionally, investigating the specific greenspace attributes influencing adult dietary intake and food choices is warranted. When devising public health interventions, it is crucial to account for the substantial health advantages associated with various socioeconomic groups.

## Graphical abstract

Four observational studies were included; results were mixed: two studies found no significant correlation between greenspace exposure and dietary patterns, while the other two indicated a positive relationship with reduced obesity and increased physical activity. The review cautions that the limited number of studies, along with socioeconomic influences on health outcomes, means that these findings should be interpreted with caution and further research is necessary to confirm these relationships and understand the mechanisms involved.

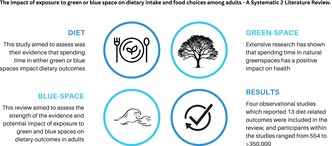

## 1 INTRODUCTION

*Greenspaces* are open areas with plants and trees that should be easily accessible to all and provide an opportunity for individuals to be active (Wolch et al., 2014). Wolch et al. (2014) observe that access to greenspace is not equitably distributed, with some neighborhoods having fewer opportunities for exposure to natural environments compared to others. Greenspace exposure or lack of can lead to a variety of health issues as people may not have areas in which to be physically active or experience mental health benefits (Wolch et al., 2014). Additionally, children who do not have greenspace exposure for play may develop behavioral difficulties (Wolch et al., 2014). Britton et al. (2018) define *bluespace* as outdoor water environments, including oceans, rivers, and lakes, which are used to promote human health and well‐being through structured therapeutic activities or programs. There is an increasing amount of literature published that highlights the advantages of spending time in greenspaces. Many individuals perceive an improvement in mental wellbeing after exposure to these environments (Stigsdotter et al., 2010; Twohig‐Bennett & Jones, 2018; van den Berg et al., 2016; White et al., 2019; Wood et al., 2017), improved weight status and obesity‐related health outcomes (Ghimire et al., 2017; Lachowycz & Jones, 2011; Luo et al., 2020), and all‐cause mortality (Burkart et al., 2016). Evidence also links greenspace to perceived general health (Kondo et al., 2018; Markevych et al., 2017; Rigolon et al., 2021). During the COVID‐19 pandemic, the time spent outdoors accessing green and blue natural spaces highlighted the beneficial effects of nature on mental health. According to a study conducted by Pouso et al. (2020), people with access to nature and greenspace during the lockdown period reported better‐coping mechanisms. Research has shown that individuals living in urban areas with greater exposure to bluespaces, such as bodies of water, tend to have better mental health outcomes (Nutsford et al., 2016). Additionally, access to safe and clean bluespaces has been linked to various positive health benefits for people, such as improved mental health and well‐being, reduced stress and anxiety, increased physical activity and exercise, enhanced mood and positive emotions, and improved cognitive function and attention restoration (White et al., 2020).

One possible reason proposed for the benefits of greenspace, is that greenspace provides more opportunities for outdoor activities, relaxation, and exercise, which can positively affect health (van den Berg et al., 2015). A theoretical framework has been created to analyze these interactions, which uses socio‐ecological theories to identify causal pathways and outcomes (Lachowycz & Jones, 2013). However, evidence on the mechanisms of these interactions remains elusive, and the results are inconsistent (Astell‐Burt et al., 2013; Fong et al., 2018; Jimenez et al., 2021; MacMillan et al., 2018; Song et al., 2019). The relationship between exposure to greenspace and improved health outcomes is confounded and mediated by many factors, including socioeconomic factors and greenspace characteristics including the quality, quantity, and use of greenspace (Freeman, 2010; Nieuwenhuijsen et al., 2019; Orioli et al., 2019; Picavet et al., 2016; Triguero‐Mas et al., 2017; Wham et al., 2012; Zandieh et al., 2019; Zijlema et al., 2020). Zandieh et al. (2019) reported that the quantity of available greenspace was significantly associated with increased walking levels in high‐poverty neighborhoods. Knobel et al. (2021) investigated the quality of urban greenspace and found that higher‐quality greenspace was associated with more frequent physical activity. However, a study by Picavet et al. (2016) found that the percentage of greenspace in the living environment was not associated with improved health benefits but did report that the distinction between greenspace types may be relevant for increased levels of physical activity. Increased levels of urban greenspace was associated with more time spent on sports, whereas increased exposure to agricultural greenspace were associated with more time spent gardening.

To date, studies regarding the possible advantages of being around natural environments and the effects of their impact on obesity have mainly focused on the influence of physical activity and the underlying mechanisms and correlations for these health benefits. How greenspace is defined in studies affects how outdoor physical activity is linked to obesity and does not focus on the additional benefits that spending time in natural environments may have for individuals with obesity (Klompmaker et al., 2018). People's dietary habits and food consumption patterns change over time. Such dietary changes are partly due to societal and environmental changes, which have led to increased consumption of energy‐dense foods. Reduced levels of physical activity and increased sedentary lifestyles have resulted in a threefold increase in obesity worldwide (World Health Organization, 2021). In fact, dietary factors are associated with five of the 10 leading causes of death, including ischaemic heart disease, cancer, stroke, and type II diabetes mellitus (Müller & Soares, 2019; Swinburn et al., 2011).

The diets of socially disadvantaged groups frequently lack nutritious items, such as fruits and vegetables (Martin et al., 2017), and with increasing urbanization, food insecurity has also increased in cities worldwide. By 2050, the United Nations predicts that over 68% of the world's population will live in urban areas (United Nations, Department of Economic and Social Affairs 2018). Sustainable urbanization, including access to greenspace and improved dietary patterns, are crucial in meeting the UN 2030 Agenda for Sustainable Development Goals to ensure that social, cultural, economic, and environmental determinants of health and well‐being are addressed (de Vries et al., 2020).

As outlined, most research to date on exposure to green and bluespaces has focused on specific health outcomes. However, the evidence is inconclusive, and the findings concerning this association are inconsistent. Given the global burden of non‐communicable diseases and the urbanization of countries, research into other mechanisms that may influence better health outcomes concerning greenspace is required. Exploring the potential effects or relationships between diet and greenspace could offer further insights.

This systematic literature review aims to examine the relationship and strength of evidence between exposure to greenspace or bluespace on dietary intake and food choices among adults. The findings of this review will provide evidence that can support and inform researchers and policymakers to develop and implement appropriate context‐specific public health interventions for improved health benefits.

## 2 METHODOLOGY

### 2.1 Search strategy

Standards as documented in the “PRISMA 2020 statement: An updated guideline for reporting systematic reviews” was followed throughout this review (Page et al., 2021). The PRISMA checklist is presented in Table S1. Details of the protocol for this systematic review were registered on PROSPERO (PROSPERO 2021 CRD42021297100) and can be accessed at the following address: https://www.crd.york.ac.uk/prospero/display_record.php?ID=CRD42021297100.

Databases were selected and agreed with the author group, which best represented health, health‐related topics, human impact on the environment, alternative medicine topics, biomedical, and life sciences. In the original systematic literature review, five electronic databases were reviewed from 1st January 2010 to 14th June 2021 to retrieve relevant articles for inclusion in the review (Cumulative Index to Nursing and Allied Health Literature (CINAHL) Complete, GreenFILE, Allied and Complementary Medicine Database (AMED), Medline, and PubMed). The electronic databases were accessed on 14th June 2021. As part of our commitment to maintaining the relevance and comprehensiveness of our findings, these searches were rerun in January 2024, following the same search strategy outlined in the PRISMA 2020 statement. This update included the same databases (CINAHL Complete, GreenFILE, AMED, Medline, and PubMed).

To construct an appropriate search strategy, previously published systematic reviews on greenspace and bluespace were searched (Britton et al., 2018; Markevych et al., 2017; Rigolon et al., 2021). The definition of greenspace was constructed for this review to utilize both quantitative and qualitative aspects of exposure to greenspace (Taylor & Hochuli, 2017). Greenspace is defined as a concept of nature, and this review included all areas of greenspace, vegetation, open space, and parklands. Bluespace was defined as either man‐made (canals), naturally occurring freshwater bodies (e.g., rivers and lakes), or saltwater bodies (oceans and seas) (Britton et al., 2018).

The search terms and definitions of green and bluespaces that formed the basis of the search strategy were identified to capture relevant articles (i.e., exposure to greenspace or bluespace and dietary outcomes).

A search string was developed for each green and bluespace concept and applied to all the electronic databases (S2). Truncation and phrase searching were used as part of the search strings to capture the nutritional and dietary outcomes (Nutri* and Diet*). The search strategy identified studies that contained at least one keyword or synonym in each string. Each concept was then combined to obtain the final search results for each database. Limiters and additional filters were applied to ensure the implementation of the review eligibility criteria outlined in Section 2.3. Specific information concerning each database is presented in the search strategy document (S3).

### 2.2 Selection process

All articles retrieved using the search strategy were imported into Rayyan (www.rayyan.ai). Duplicate articles were identified and deleted. One reviewer (CG) independently screened titles and abstracts. A second reviewer (AS) screened titles and abstracts independently to ensure that the inclusion and exclusion criteria were functioning and being applied correctly. When there were disagreements, full‐text articles were retrieved. For the updated searches conducted in 2024, title and abstract screening was conducted by AS and AT independently. A third reviewer was not required; there were no disagreements on selected studies.

Full‐text screening was then conducted independently by one reviewer (CG) of the remaining articles using the inclusion and PICO criteria.

*Population*: Male and Female adults >18 years.

*Intervention*: Exposure to greenspace or bluespace.

*Comparison*: There was no comparator restrictor.

*Outcome*: Study reports a diet related outcome.

Articles were screened against this criterion by a second reviewer (AS), and any disagreements in the selection of articles were resolved through discussion. For the updated screening, AS conducted title, abstract, and full text screening, and AT was the updated second reviewer, ensuring a consistent and accurate application of the selection process. Studies were not included if the outcome of interest was not reported, and the reasons for excluding articles were documented in the PICO Research Checklist (Table S4).

### 2.3 Eligibility criteria

The inclusion and exclusion criteria were specified for the review and agreed with the authors' group **(**Table 1 **).** Empirical studies in which dietary outcomes were attributable to exposure to greenspace or bluespace were included. Dietary outcomes were identified as dietary intake or food choices among adults, which was essential for inclusion. The study population included adults (>18 years). Only studies that included human participants were included, and children or adolescents (<18 years) were excluded. This review included quantitative studies with both experimental and observational designs. Studies that were not peer‐reviewed were excluded.

**TABLE 1:** Inclusion and exclusion criteria.

Inclusion criteria	Exclusion criteria
Experimental or observational research testing the relationships between greenspace and bluespace and dietary outcomes	Studies that do not include experimental or observational evidence
Study reports a dietary outcome	Studies not reporting a dietary outcome
Only studies written in the English language are included	Studies written in any other language
Population in the study are adults (>18 years)	Population in the study are not adults (<18 years)
Studies must be based on human participants	Studies that do not use human participants
Studies published since 2010	Studies published before 2010

The primary aim of this review is to examine the relationship between exposure to greenspace or bluespace and dietary outcomes, including studies that incorporated outcomes concerning food practices and food habits. Studies that demonstrated diet, eating habits, and dietary patterns, which were influenced or managed in statistical analysis and reported multiple results, should be included to help further explain the complex interactions and causal relationship between exposure to the intervention and the outcome.

Only studies written in English were included because of a lack of translational capacity. As most of the research on green and bluespace interventions has been published in the last 10 years, the review was restricted to studies published from 2010 onwards.

### 2.4 Data collection process and data items

Data extraction was performed for all eligible full‐text articles. Owing to the limited number of articles in the review, articles were grouped for synthesis based on the outcome of interest. The following information was extracted: author, title, year of publication, country, study design, participants, intervention, measurement tool used, outcome under investigation (food choices/dietary intake), and other outcomes/notes. Any disagreements in data collection were discussed and, if necessary, reviewed by a third investigator (EMOC or AT).

Where multiple results were available, the outcome considered most important for this review (dietary outcome) was recorded, and other outcomes were documented separately during the data extraction process.

### 2.5 Critical appraisal

Two authors conducted rigorous critical appraisals of the included studies. The Joanna Briggs Institute (JBI) Critical Appraisal Checklist for Analytical Cross‐Sectional Studies is recommended as the preferred tool for assessing the quality of analytical cross‐sectional studies (Ma et al., 2020). The (JBI) Critical Appraisal Checklist is a domain‐based tool that includes eight criteria: three referring to the study population, one criterion related to validity and reliability concerning the measurement of exposure, one criterion related to validity and reliability concerning the measurement of outcomes, and three criteria related to statistical analysis and adjustment for confounding factors (Moola et al., 2020). A criterion was rated Yes if enough information was available to determine the criterion was met, No if it was not met, Unclear if a decision could not be applied with the information provided, and Not applicable if the criterion did not apply. The sum of all the positive scores was added to determine the overall quality score. A score of 100% indicated that the study was of high quality (HQ), and a score of 70–100% was rated medium quality (MQ). If a study scored <70% on the critical appraisal, it was deemed low quality (LQ). A critical appraisal of all the included articles was conducted independently by one reviewer (CG) and checked by a second reviewer (AS). Disagreements were resolved through discussion and referral to the full text of the article if required. Critical appraisal checklists for all included articles are available in Data S5. As no additional studies met the inclusion criteria after the updated search, critical appraisal was not necessary.

### 2.6 Narrative synthesis

Owing to the heterogeneity of greenspace measures and dietary outcomes, a meta‐analysis approach was not pertinent. A narrative synthesis using synthesis without meta‐analysis (SWiM) reporting guidelines was conducted for this review (Campbell et al., 2020). The synthesis covered how studies were grouped, synthesis methods used, presentation of data, and limitations of the synthesis. Owing to the limited number of studies included in the review, full‐text articles were grouped for narrative synthesis based on the outcome of interest **(**Table 2 **)**, and standardized metrics for each outcome are reported in Table 3. To extract the data, thematic analysis was used to identify the main, recurrent, and most important themes and concepts across studies. Establishing a common rubric for the included studies was not considered useful as there was no unit of measurement that could form the basis of a common rubric due to the heterogeneity of measurements of intervention and outcomes. Due to the limited number of articles included in the review, specific criteria were not used to prioritize the reporting of some study findings over others, and the synthesis was not restricted to a subset of studies. An assessment of the certainty of evidence was not performed because of the limited number of studies included in the review. Study findings are presented in the same way as synthesis to facilitate the comparison of findings from each included study. Each outcome is described in the synthesis along with the limitations associated with each study.

**TABLE 2:** Study characteristics of included studies.

Study ID		Population			Intervention			Outcomes			Societal factors
Author/year	Study design	Country	Sample size	Gender and age	Green space/bluespace	Measurement of greenspace intervention	Measurement of dietary outcome	Dietary intake/food choice	BMI	Physical activity	SES
Yuen et al. (2019)	Cross sectional study	Hong Kong	554	Male:198 Female: 356 Age 48.05 years ± 20.98 years	Greenspace	% of greenspace within a 500 m radius buffer around each participant's residence calculated using SPOT satellite image‐derived data set	Four‐part Likert scale self‐reported by participants	Low sugar Low salt Low fat High fruit	Not reported	IPAQ‐C MET –mins/week calculated	Monthly income Education
von Hippel and Benson (2014)	Cross sectional study	United States	350,000	>20 years	Green space/Bluespace	Percentage of the county area that is covered by forest or water, and topographic relief measured on a scale that runs from 1 for “flat plains” to 21 for “high mountains. This is based on the natural environment scale in the study	BRFSS, Behavioral Risk Factor Surveillance System (BMI > 30) Self‐reported To measure dietary patterns data from US Department of Agriculture's Food Environment Atlas retrieved	Soda Sweet/snacks Prepared foods Solid fats Meat	The percentage of county residents aged 20 years and older whose BMI exceeded 30	% physical activity	–
Choi and Yoon (2020)	Case control study	South Korea	577	Samples were between 19 and 64 years old; 38.1% are male; 61.9% are female	Greenspace	Built environment data were collected from a number of sources, including National Geographic Information Institute, Statistical Geographic Information Service, Korea Forest Service, and Seoul Metropolitan Government	KNHNES (Korea National Health and Nutrition Examination Survey) for BMI data on participants; Construct on Dietary patterns developed for analysis	Eat out Carbohydrate ProteinFat Sodium	BMI is the main dependent variable	Frequency of weekly and daily physical activity	Education Monthly income
Michimi and Wimberly (2012)	Cross sectional study	United States	457,820 participants for obesity and 473,296 for physical activity	Adults aged over 18 years (48% males; 52% females)	Greenspace	Recreational and natural amenities indices based on measurements of physiography and cover	BRFSS, Behavioral Risk Factor Surveillance System (BMI >30) Self‐reported; Tourism variable to capture food services industry; data from US Census County Business Patterns Dataset 2000	Outdoor activity potential indices highlights natural environments reduce obesity and increase physical activity	Height and weight to calculate BMI, used in obesity model	Odds Ratios for Physical Activity OR 1.08 (95% CI 1.07, 1.10)	Education Income

**TABLE 3:** Study results.

Study ID	Results				Setting
	Dietary outcomes	Body mass index (BMI)	Physical activity	Socio‐economic status (SES)	
Yuen et al. (2019)	Low sugar 0.051 Low salt 0.071 Low fat 0.084 High fruit 0.069 *Note*: *Correlational analysis among variables measured in the studied population*, *p‐value*; Table 3 Education *p* < .001; Age *p* < .001	–	MET – minutes/week increased with increased green space (*p* < .031) *Note*: *Physical activities and dietary habits of participants living with different greenspace levels*; Table 2	Education 0.227 Monthly income 0.002 *Note*: *Correlational analysis among variables measured in the studied population*, *p‐value*; Table 3	*Parks*, *promenade*, *and sports facilities*
von Hippel and Benson (2014)	Soda −0.065 (−0.262, −1.851) Sweet/snacks −0.165 (−0.389, 0.060) Prepared foods 0.320**(0.092, 05.48) Solid fats 0.149 (−0.030, 0.328) Meat 0.206**(0.065, 0.348) *Note*: *Multiple Regression predicting county obesity with physical activity and food*, *b* (*95% CI*); Table 2	% obese 18–52%; SD 4% Strong negative correlation between obesity and physical activity (*r* = −0.83) Exposure to bluespace/greenspace; no significant association with obesity (*p* < .1) Weather variables with significant association with obesity, extreme heat, cold, and dark rainy conditions *p* < .05	% physical activity 32–74%; SD 6%	–	*Natural environment*
Choi and Yoon (2020)	Eat out 1.000 Carbohydrate 1.200*** Fat 1.319*** Sodium −0.329*** *Note*: *SEM Analysis of how neighborhood environments effects BMI*; *Figure 4* ***p < .01; **p < .05; *p < .1	**Dependent variable** Walkability −3.385** SES −9.091** Urban Leisure 1.069** Dietary Pattern 0.700** *Note*: *SEM Analysis of how neighborhood environments effect BMI*; *Figure 4*	Weekly vigorous work 1.000 Weekly moderate work 0.259*** Daily vigorous work 1.050*** Daily moderate work 0470*** Weekly vigorous recreation 0.002 Weekly moderate recreation −0.001 Daily vigorous recreation −0.069 Daily moderate recreation 0.080 Weekly active commuting 0.083 Daily active commuting 0.101** Weekly walking 0.037 *Note*: *SEM Analysis of how neighborhood environments effects BMI*; *Figure 4*	Education 1.000 Monthly income 1.498*** SES to Walkability −0.947*** SES to Urban Leisure 1.162*** *Note*: *SEM Analysis of how neighborhood environments effects BMI*; *Figure 4*	*Built environment*
Michimi and Wimberly (2012)	Tourism variable classified by NAICS	Odds Ratios for obesity OR 0.91 (95% CI 0.90, 0.93) 9% reduction in the odds ratio for obesity with a 1‐unit increase in the natural amenities index for outdoor activity potential	Odds Ratios for Physical Activity OR 1.08 (95% CI 1.07, 1.10) Physical activity increased with increasing education and income in both recreational opportunity index and natural amenities index *Note*: *Table 3 Odds Ration for Physical Activity with OAP indices*	Obesity decreased with increasing education and income in both recreational opportunity index and natural amenities index *Note*: *Table 2 Odds Ration for Obesity with OAP indices*	*Natural amenities*

* *p* < 0.1: The result is statistically significant at the 10% level, meaning there is less than a 10% chance that the observed relationship occurred by random chance.

** *p* < 0.05: The result is statistically significant at the 5% level, meaning there is less than a 5% chance that the observed relationship occurred by random chance.

*** *p* < .01: The result is statistically significant at the 1% level, meaning there is less than a 1% chance that the observed relationship occurred by random chance.

## 3 RESULTS

### 3.1 Study selection

**FIGURE 1:**
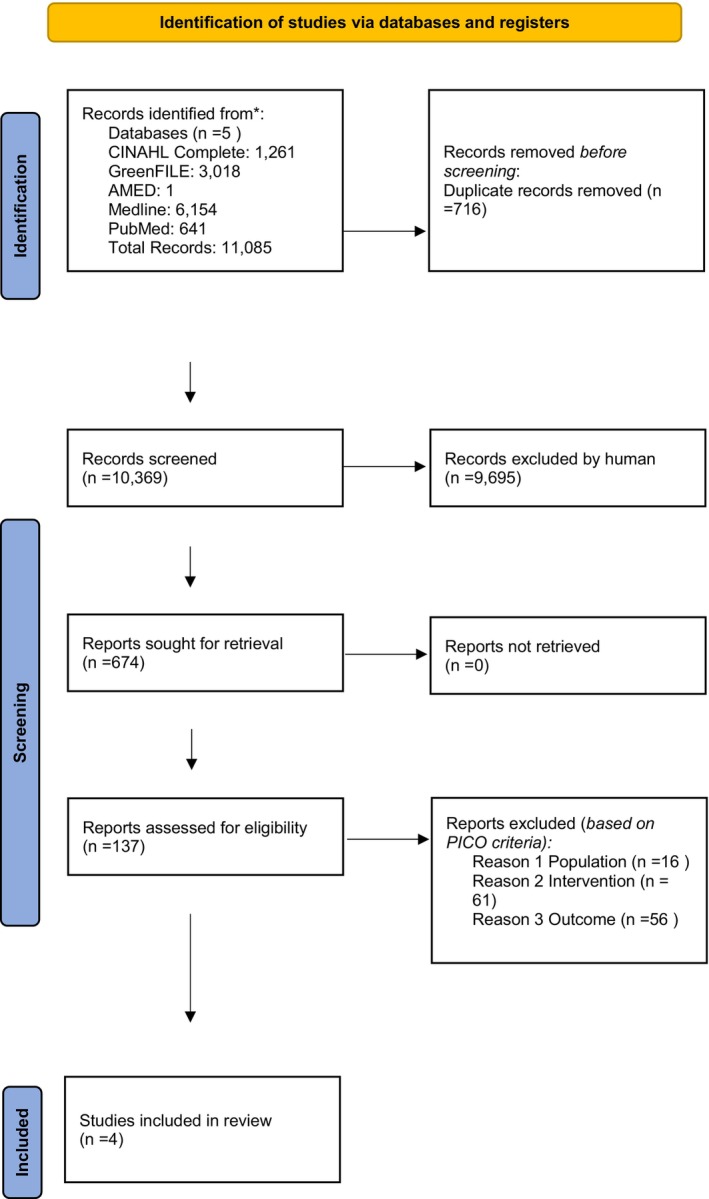
PRISMA 2020 flow chart of selected studies. *means reporting the number of records identified from each database or register searched (rather than the total number across all databases/registers), as seen on Prisma flow chart.

A total of 10,897 records were identified through an electronic database search. After removing duplicates, 10,181 records were screened, of which 666 were retrieved for abstract screening. A total of 137 articles were read as full text for eligibility. Four studies were deemed eligible for inclusion after an independent assessment by a second reviewer (AS). The flowchart is shown in Figure 1. Following the updated search in January 2024, which aimed to identify articles published after the initial review period and incorporate the newly identified database, the search yielded an additional 188 papers.

Several studies were retrieved from the original search concerning bluespace. However, most of these studies did not specify a link to the outcome or the intervention and were mostly related to the eutrophication of urban and rural waterways and their nutrient content. Several other studies on bluespace have reported exposure to persistent pollutants, consumption of fish, and the assessment of drinking water quality (Baho et al., 2019; Bawa et al., 2018; Zhou et al., 2016, 2017). Reasons for exclusion of all studies are documented in the detailed PICO Research Checklist Table S4.

### 3.2 Study characteristics

After a comprehensive search and rerun in January 2024, no additional studies were found for inclusion. Although a date restriction was put on the search, 3 out of 4 of the articles reviewed at the full‐text stage were published within the last 6 years. Two of the four included studies were conducted in the United States (Michimi & Wimberly, 2012; von Hippel & Benson, 2014), one in South Korea (Choi & Yoon, 2020) and one in Hong Kong (Yuen et al., 2019). All studies were observational, three were cross‐sectional, and the fourth was a case–control study. The sample sizes under investigation varied across the four studies, and two studies used routinely collected data from the Behavioral Risk Factor Surveillance System (BRFSS) in the United States, accessing sample sizes of >350,000 surveyed participants. One study used primary data collection from 554 residents, and the fourth study accessed the National Health and Nutrition Examination Survey in Korea (KNHNES) and included 577 survey responses.

Four different types of exposure to greenspaces were measured in studies using a number of different measurement tools to assess exposure. In one study, the percentage of greenspace within a 500 m buffer around each participant's home was calculated using SPOT satellite imagery (Yuen et al., 2019). A second study (von Hippel & Benson, 2014) calculated the percentage of the county area under investigation covered by forest and water. Information and data on forest cover were obtained from the US Forest Service, and data on water cover were obtained from the Economic Research Service of the US Department of Agriculture. A third study (Choi & Yoon, 2020) used data on built neighborhood environments represented by walkability and leisure amenities collected from various sources, including the National Geographic Information Institute, Statistical Geographic Information Services (SGIS), Korea Forest Service, and Seoul Metropolitan Government. The final study in the review (Michimi & Wimberly, 2012) measured exposure to greenspace at the county level using two indices of outdoor activity potential (OAP). These two indices include a recreational opportunity index and a natural amenities index. The recreational opportunity index was calculated using data from outdoor recreational facilities. Data from the USDA Forest Service Southern Research Station were obtained from the 1997 National Outdoor Recreation Supply Information System (NORSIS). The natural amenities index was created using data on physiography, cover, climate, and tourism employment.

Dietary and other health outcomes, including physical activity and obesity, were reported in each study. The characteristics of the studies are listed in Table 2. The dietary intake methodology for the study outcomes is described below.

### 3.3 Study outcomes

The measurement of dietary outcomes varied across the four included studies, with three studies controlling for diet in their analysis. One study measured healthy eating practice on a four‐part Likert scale, self‐reported by the participants (Yuen et al., 2019). Three studies reviewed the adult obesity prevalence in different settings using statistical analysis, mediating or controlling for diet within the analysis. One study retrieved five diet‐related variables from the US Department of Agriculture's Food Environment Atlas (von Hippel & Benson, 2014). Another study developed a construct on dietary patterns using several different indicators (Choi & Yoon, 2020) with data retrieved from a national health and nutrition survey. The final study captured information on the food services industry using data from the US Census County Business Patterns Dataset 2000 (Michimi & Wimberly, 2012). Two of the studies also assessed measurements of body mass index (BMI) from the National Behavioral Risk Factor Surveillance System (BRFSS) (Michimi & Wimberly, 2012; von Hippel & Benson, 2014).

All studies in this review investigated more than one outcome, identifying the interrelationship between dietary choices, greenspace, and other health outcomes. The reported dietary outcomes included dietary intake and food choices. Physical activity and obesity have been reported to be separate health outcomes.

However, the results varied between studies (Table 3). Healthy eating habits were not directly linked to exposure to greenspace in one study (Yuen et al., 2019), and the results were not statistically significant for a number of dietary intakes, including low sugar (*p* < .051), low salt (*p* < .071), low fat (*p* < .084), and high fruit intake (*p* < .069). This study identified that dietary intake and food choice were correlated with other demographic and quality of life (QoL) variables and also identified a positive association between greenspace and physical activity (*p* < .031). Yuen et al. (2019) examined how exposure to greenspaces (like parks) affects food choices. According to the authors, exposure to greenspace by volume influences dietary choice: people living with a lot or a little greenspace around them usually did not eat much fat, salt, or sugar and ate lots of vegetables and fruits. However, people with moderate greenspace exposure eat less healthily (Yuen et al., 2019). The “medium greenspace” subgroup had the highest percentage of participants who consumed less than one serving of vegetables (58%) and fruits (40%), less than one serving of vegetables daily (Yuen et al., 2019). About 25–30% of these individuals rarely choose foods low in fat, salt, or sugar. Unlike exercise, living near greenspaces is not linked to eating healthily, with the exception of people living in more greenspaces who eat fewer vegetables (=quality of life 0.087; *p* = .041; Yuen et al., 2019).

Two further studies (Michimi & Wimberly, 2012; von Hippel & Benson, 2014) reviewed obesity and the role of the natural environment on obesity outcomes, controlling for dietary patterns in the analysis, where both identified an association between these outcomes. von Hippel and Benson (2014) looked at if exposure to the natural environment affects how much people weigh by changing what they eat and found that exposure to green/bluespace did not significantly change their eating habits. They also used information about how much soda, snacks, and other foods people bought to see if that could explain clustering of overweight people in specific places; however, physical activity appeared to have a bigger impact. These authors hypothesized that the effect of the natural environment on obesity is mediated by diet. To test this, they used five variables from the US Department of Agriculture's Food Environment Atlas measuring each county's per capita purchases of soda, packaged sweet snacks, prepared foods, solid fats, meats, and fruits and vegetables for home consumption. They identified that exposure to green and bluespaces, as measured by percentage water area and forest cover, had no significant association with obesity (*p* < .1), with or without food as a mediator in the analysis. However, this study also identified a strong negative relationship between obesity and physical activity (*R* = −0.83). Michimi and Wimberly (2012) identified a positive correlation between obesity and the natural environment, reporting a 9% reduction in the odds ratio for obesity with a 1‐unit increase in the natural amenities index for outdoor activity potential (OR, 0.91; 95% CI, 0.90, 0.93), indicating that increased access to natural environments and outdoor activity opportunities was associated with lower odds of obesity in the populations studied.

The final study in the review (Choi & Yoon, 2020) when controlling for dietary patterns, also reported the association between obesity and the built environment, quantified through walkability in neighborhoods and access to leisure amenities. The dietary pattern construct measured individuals' dietary habits and was constructed using the following indicators: the number of times individuals eat out per week (eat out) and the total daily intake of carbohydrate (carbohydrate), protein (protein), fat (fat), and sodium (sodium). Choi and Yoon (2020) recorded dietary patterns for carbohydrates, fats, sodium, and eating out and their relationship with exposure to the natural environment or factor loading, representing this relationship's strength. The result showed that exposure to green/bluespace had a significant effect on all dietary patterns except carbohydrates (*p* < .01). Additionally, the authors noted that the frequency of eating out may indicate socioeconomic status and poorer access and exposure to amenities such as parks. Furthermore, this study reported a negative association between BMI and walkability (*p* < .05) indicating that a higher neighborhood walkability explained lower BMI levels among residents, as expected (Choi & Yoon, 2020).

Two studies in the review identified other findings concerning the association between obesity and the natural environment. One study (Choi & Yoon, 2020) found that greater access to leisure amenities in a neighborhood did not negatively affect BMI but were, in fact, associated with an increase in BMI (coeff = 1.069). This study highlighted that although people have access to leisure amenities, including open spaces in their neighborhood, this does not mean that people will use them. von Hippel and Benson (2014) reported that prevailing weather conditions, where extreme heat, cold, and dark rainy conditions increased obesity with the coefficients of several variables from January sunlight −0.409** (−0.684, −0.134) and annual snowfall −0.238* (−0.470, −0.006), which were statistically significant for obesity (*p* < .05). Each coefficient represents the expected increase in obesity prevalence associated with a 1 standard deviation increase in the natural environment variable being examined. The association between obesity and exposure to the natural environment, including trees, waterfronts, hills, and mountains, was not statistically significant.

Socioeconomic status (SES) was also reported in two studies, with results from Yuen et al. (2019) indicating that dietary intake and food choices were correlated with different demographic and WHO QoL variables, including education level (*p* < .001) and age (*p* < .001). According to this study, lower socioeconomic groups benefitted more from green areas in their urban environments (Yuen et al., 2019). A second study (Choi & Yoon, 2020) reported a statistically significant effect of SES on BMI (*p* < .05). SES also had a statistically significant impact on walkability (*p* < .01) and urban leisure (*p* < .01). Furthermore, SES showed a statistically insignificant relationship with dietary pattern.

### 3.4 Critical appraisal

All four articles in the review were critically assessed for quality using the Joanna Briggs Institute (JBI) Critical Appraisal Checklist for Analytical Cross‐Sectional Studies (S5). Two studies were deemed to be of high quality (HQ) (Choi & Yoon, 2020; von Hippel & Benson, 2014) and two studies were deemed to be of low quality (LQ) (Michimi & Wimberly, 2012; Yuen et al., 2019). However, none of the studies were excluded because of low‐quality scores. Two studies deemed to be of low quality scored 63%, scoring five out of eight criteria. Two high‐quality studies scored eight out of eight. The two checklist criteria missing from both studies concerned confounding factors and strategies for dealing with confounding factors. Individual quality analysis scores are shown in Data S5.

## 4 DISCUSSION

### 4.1 Summary of findings

This study aimed to assess the potential impact of exposure to greenspace and bluespace on the dietary intake and food choices of adults. Several studies have investigated the association between greenspace and different health outcomes, including improved mental health (Annerstedt et al., 2012; Triguero‐Mas et al., 2017; Britton et al., 2018: Korn et al., 2018; Lee & Lee, 2019), reduced prevalence of type 2 diabetes (Burkart et al., 2016; Teufel‐Shone et al., 2014), reduced mortality (Burkart et al., 2016; Orioli et al., 2019), and improved pregnancy outcomes (Abelt & McLafferty, 2017). However, this is the first review to examine changes in dietary patterns following exposure to green and bluespaces, and no conclusive data were found on the association between them.

The first significant finding of this review was the lack of evidence and studies available concerning bluespace. A small number of articles were retrieved; however, none met the inclusion criteria (Britton et al., 2018; Burkart et al., 2016; Goeminne et al., 2015). Further research should be conducted on the potential health benefits of bluespace on dietary patterns, changes in dietary habits, and the associated health benefits.

In contrast, although greenspace is well researched, limited evidence is available on exposure to greenspace and impact or changes to dietary patterns, and only four studies were included in this review. Interpreting the results is further complicated because of the heterogeneity of the documented interventions and outcomes.

However, despite the limited findings in this review, emerging evidence demonstrates that implementing urban horticulture can significantly improve urban biodiversity and population health. Marginalized communities stand to gain the most from urban horticulture, which in recent studies positively impact health, promote healthy food intake, exercise, nature exposure, and community social cohesion (Algert et al., 2014; Clendenning et al., 2015; Martin et al., 2017). Further research is required to enhance the current evidence regarding the positive impact on human health (Cruz‐ Piedrahita et al., 2020).

The four observational studies included in this review identified an association between greenspace exposure and obesity. Yuen et al. (2019) indicated that exposure to greenspace was not correlated directly with healthy eating patterns but with physical activity levels. This finding is consistent with that of von Hippel and Benson (2014), who found that diet had no effect on the association between obesity and the natural environment and that the relationship was mediated by physical activity. A similar finding was also reported by Michimi and Wimberly (2012), who discovered that as recreational opportunities and natural amenities increased, physical activity levels increased, and the prevalence of obesity decreased. An association between obesity and physical activity was also identified in the final study conducted by Choi & Yoon, 2020, in which a negative association between BMI and walkability in urban neighborhoods was identified. While the main research question addressed in this review explicitly concerns dietary pattern changes, secondary outcomes concerning exposure to greenspace and obesity were addressed.

### 4.2 Greenspace and obesity

The health benefits of exposure to greenspace and obesity have been well researched; however, the mechanisms and pathways underlying these associations are complex. Obesity is a complex disease, and several factors play a role in gaining weight. However, one of the significant causes of obesity (BMI > 30) and overweight (BMI > 25) is the consumption of excessive energy‐dense foods combined with a lack of exercise (World Health Organization, 2021). One of the potential mechanisms explored to explain the association between greenspace and health outcomes is the accessibility and availability of greenspace, which may influence individuals' physical activity levels and subsequently, weight status. Yuen et al. (2019) found that physical activity levels were positively related to the percentage of greenspace and accessibility of open space facilities. In a study by von Hippel and Benson (2014), obesity was reported to be more prevalent in hot, dark, cold, and rainy counties in the US. These findings raise the possibility that individuals within these counties, owing to adverse weather conditions, are not engaging in outdoor physical activity, resulting in an increased prevalence of obesity. Attempts to promote physical activity should consider temperature extremes. Although little is known about how exercise affects dietary habits, evidence suggests that exercise motivates people to adopt healthier eating habits (Joo et al., 2019). Thus, moderate physical activity can serve as a gateway behavior, leading to the adoption of other healthy habits (Blakely et al., 2004; Grant et al., 2020; Lounassalo et al., 2021).

Previous studies in this area have noted positive associations between greenspace and obesity‐related indicators, including physical activity, BMI, and obesity‐related health outcomes (Villeneuve, 2018; Huang et al., 2020; De la Fuente, 2021). According to a systematic review (Luo et al., 2020), there may be a link between increased access to greenspace and a lower risk of obesity. The results of the meta‐analysis showed that individuals exposed to increased levels of NDVI (Normalized difference vegetation index) were less likely to be overweight/obese (OR: 0.88; 95% CI: 0.84, 0.91); however, the review also identified that further high‐quality studies are required to assess the evidence for this causal relationship. These findings are consistent with an earlier systematic review, which found a positive link between greenspace and obesity but mixed evidence on the association between greenspace access and physical activity (Lachowycz & Jones, 2011). A study in the US reported lower BMI levels in individuals exposed to increased forest cover, and this association was stronger for those who participated in outdoor recreation (Ghimire et al., 2017). These studies reinforce the connection between opportunities for physical activity and a lower prevalence of obesity.

However, the results concerning greenspace exposure and obesity have been mixed across studies. One interesting finding was that more urban leisure amenities in a neighborhood were associated with higher BMI levels (Choi & Yoon, 2020). This finding suggests that promoting walkability in neighborhoods may be more successful in targeting obesity than leisure amenities.

Another interesting finding was that exposure to natural elements, such as wind, trees, waterfronts, and mountains, had little or no association with obesity (von Hippel & Benson, 2014). Recent Irish research (Dempsey et al., 2018) discovered evidence of a U‐shaped relationship between obesity and greenspace. The study identified that people living in the highest and lowest areas assessed for greenspace had higher probabilities of obesity. These preliminary findings suggest that even though individuals may have access to greenspaces and other amenities in their neighborhood, it does not mean they will avail of them to promote health. These relationships may partly be explained by other confounding and mediating factors, for example, access to eateries, which may have the opposite effect to the intended effect (Lovasi, 2009). In other words, the association between the natural environment and obesity appears to be nuanced and non‐linear, potentially influenced by a variety of contextual elements beyond just the presence of greenspaces. Further research is needed to elucidate the mechanisms underlying these mixed findings.

Healthy eating habits also moderate obesity. However, this study discovered that healthy eating habits were not related to greenspace but rather to other demographic and quality of life (QoL) variables, such as education and age (Yuen et al., 2019). Again, this finding is consistent with the literature, and several factors are associated with food choices, including socioeconomic and demographic factors and social mobility (Arruda et al., 2014).

More research is required to examine how dietary habits and physical activity impact exposure to greenspaces to establish a more robust correlation and identify any causal links. In addition, health interventions need to focus on promoting healthy behaviors such as maintaining a healthy diet and engaging in physical activity. Studies have shown that individuals who make one positive behavioral change are more likely to make additional changes successfully (Lippke et al., 2011). It is important to examine the characteristics of the physical surroundings since studies suggest that easy access to walking paths may have a stronger correlation with obesity rates than other aspects of city or environmental facilities. Health interventions should consider temperature extremes as they are not conducive to outdoor physical activity.

### 4.3 Greenspace and socio‐economic status (SES)

Evidence suggests that a person's socioeconomic status (SES) is crucial in determining their food choices and the likelihood of obesity (Thorpe et al., 2019; Wham et al., 2015). Mounting evidence suggests that exposure to greenspace may be “equigenic” (Mitchell et al., 2015), meaning that individuals in lower socio‐economic groups may experience the most significant benefits from such exposure. This finding was reported by Yuen et al. (2019), who identified that lower socioeconomic groups, such as the elderly, youth, and the less educated, appeared to benefit more from greenspace in their living urban environment. A similar finding was identified in a study by Maas (2006), who reported that percentage greenspace within one and three kilometers radius was significantly related to improved self‐perceived general health by individuals. This positive association was stronger for lower socioeconomic groups, including the elderly, youth, and second‐level education. These results are consistent with those of Choi and Yoon (2020), who reported that SES has a negative effect on BMI and identified that the impact of SES is much more significant on obesity than physical activity and access to environmental amenities. Similarly, Rigolon et al. (2021) discovered that greenspace has greater health benefits in low‐income countries, and public greenspace has a greater protective effect for lower SES groups. There is a strong link between obesity and SES. According to a cross‐sectional study conducted in the United States (Wen et al., 2017), the rates of obesity were found to be higher in rural areas and associated with education level and median household income. Thorpe et al. (2019) found that a lower SES and education were predictors of more deficient dietary patterns.

Other characteristics of greenspaces, including accessibility, quality, size, and facilities for certain activities, offer different opportunities for physical activity and other health benefits for individuals. Further research is necessary to determine the characteristics required for specific health outcomes. Individual user determinants are also crucial, including age, gender, ethnicity, and safety when using greenspace, and these should also be taken into account.

### 4.4 Limitations and strengths of the study

The strengths of this study include the broad inclusion criteria used for greenspace and bluespace and their effects on dietary outcomes to ensure that all relevant data were considered. Studies were not excluded based on the study design, type of greenspace, or measurement of exposure to greenspace. Consequently, a variety of greenspace exposures and outcomes were identified. However, this can also result in high heterogeneity across studies and difficulties in comparing and interpreting study results. Despite the inclusivity of this review, only a few articles that met the inclusion criteria were retrieved. Therefore, these findings should be cautiously interpreted. This review demonstrates the lack of research in this area, and further studies on dietary patterns, including food choices and dietary intake, should be undertaken.

Another limitation of this study was its methodological design. All included studies were observational, with a cross‐sectional design being the most common. This review emphasizes the need for longitudinal and experimental research in this area. In observational studies, there is a potential for bias, including selection bias, information bias, and confounding. In two of the studies used in this review (Michimi & Wimberly, 2012; von Hippel & Benson, 2014), BMI measurements for participants were based on self‐reported heights and weights collected by the Behavioral Risk Factor Surveillance System (BRFSS), a telephone survey of more than 350,000 US adults per year. As this information is self‐reported, there is a risk of over‐ and under‐reporting and compromised objectivity. To correct for self‐reported bias in one study (von Hippel & Benson, 2014), newly released bias‐corrected estimates of county obesity prevalence were used.

Further bias can be introduced through confounding, and in two studies, quality appraisals (Michimi & Wimberly, 2012; Yuen et al., 2019), it is unclear whether confounding factors were identified and strategies were implemented to deal with these. This can result in a biased estimate of the outcomes. A third study within the review (von Hippel & Benson, 2014) identified that the associations reported in the study might be attributable to confounding factors that were not controlled for, and reverse causality is also possible. Due to complex associations and limited evidence on causal pathways for specific health outcomes in greenspaces, many confounding factors are possible. Strategies to avoid potential sources of bias should be adequately considered by careful planning, and future research should consider this.

## 5 CONCLUSION

This systematic review provides an updated overview of studies examining the associations between exposure to green and bluespaces and dietary intake and food choices among adults. This extensive review showed minimal evidence for an association between greenspace exposure and dietary patterns among adults. The most significant finding of this review is the paucity of evidence concerning this association, indicating that further research is required to understand the complex mechanisms involved and guide future interventions. Further research is needed to ascertain the determinants of greenspace required for specific health outcomes, including improved dietary intake and food choices among adults. The development of public health interventions concerning exposure to green or bluespaces with dietary outcomes should also consider the socio‐economic status and substantial health benefits of this group.

## AUTHOR CONTRIBUTIONS

**Claire A. Gilbourne:** Data curation (equal); formal analysis (equal); investigation (equal); methodology (equal); writing – original draft (lead). **Alan Scarry:** Formal analysis (equal); investigation (equal); methodology (equal); writing – original draft (supporting). **Eibhlís M. O'Connor:** Conceptualization (equal); funding acquisition (lead); resources (lead); supervision (lead); writing – original draft (supporting). **Audrey C. Tierney:** Conceptualization (supporting); resources (supporting); software (supporting); supervision (supporting); validation (supporting); writing – original draft (supporting).

## FUNDING INFORMATION

This research received no specific grants from any funding agency, commercial, or not‐for‐profit sectors.

## CONFLICT OF INTEREST STATEMENT

The authors declare that they have no known competing financial interests or personal relationships that could have appeared to influence the work reported in this paper.

## Supporting information


Data S1:

## Data Availability

The following supporting information can be downloaded at: https: DOI 10.17605/OSF.IO/NCJDQ: PRISMA (Preferred Reporting Items for Systematic reviews and Meta‐Analysis); Table S2: Search string used for this review; Table S3: Search terms used for this review; and Table S5 critical appraisal for this review.
