# Supplementary material for: The impact of exposure to green or bluespace on dietary intake and food choices among adults—A systematic literature review
Source: Food Sci Nutr. 2024 Nov 4;12(12):9913–27. doi: 10.1002/fsn3.4447 (PMC11666982; doi:10.1002/fsn3.4447)
Supplement: Supplementary file 1 — Data S1: [file FSN3-12-9913-s001.zip › S2 Search string.docx]

| # | *Search string* | *Description of keywords* | *Additional Filters* |
| --- | --- | --- | --- |
| S001 | Greenspace | green space OR greenspace OR greenness OR greenery OR greener | Humans, All fields, English Adults 19+ |
| S002 | Vegetation | tree OR forest OR vegetation OR woodland | Humans, All fields, English Adults 19+ |
| S003 | Open space | wild space OR open space OR public space OR natural space OR wild land OR open land OR public land OR natural land OR wild area OR open area OR public area OR natural area OR natural environment | Humans, All fields, English Adults 19+ |
| S004 | Parklands | park OR garden OR parklands OR green zones OR greenways | Humans, All fields, English Adults 19+ |
| S005 | **[Green space]** | **S001 OR S002 OR S003 OR S004** | Humans, All fields, English Adults 19+ |
| S006 | Bluespace | Blue space | Humans, All fields, English Adults 19+ |
| S007 | Surface waters | natural surface waters OR surface waterbodies OR lakes OR ocean OR sea | Humans, All fields, English Adults 19+ |
| S008 | Water courses | watercourses OR rivers OR canals | Humans, All fields, English Adults 19+ |
| S009 | **[Blue Space]** | **S006 OR S007 OR S008** | Humans, All fields, English Adults 19+ |
| S010 | Diet or diet* | Diet or diet* | Humans, All fields, English Adults 19+ |
| S011 | **Nutrition or Nutri*** | **Nutrition or Nutri*** | Humans, All fields, English Adults 19+ |
| S012 | **Food choices** | Food choices OR dietary practices OR food practices OR food consumption patterns OR food intake patterns OR food habits OR dietary habits | Humans, All fields, English Adults 19+ |
| S013 | **[Dietary Outcomes]** | **S010 OR S011 OR S012** | Humans, All fields, English Adults 19+ |
| S014 | [Green space] S005 AND [Blue Space] S009 AND [Dietary Outcomes] S013 | S005 AND S009 AND S013 | Humans, All fields, English Adults 19+ |

***S2: Sample search string for electronic searching of agreed databases***
