# Supplementary material for: The impact of exposure to green or bluespace on dietary intake and food choices among adults—A systematic literature review
Source: Food Sci Nutr. 2024 Nov 4;12(12):9913–27. doi: 10.1002/fsn3.4447 (PMC11666982; doi:10.1002/fsn3.4447)
Supplement: Supplementary file 1 — Data S1: [file FSN3-12-9913-s001.zip › S3 Search_Strategy_Documentation_Template__12_.docx]

June 15, 2021

_ _

**Claire Gilbourne**

**Public Health Research BM6043**

Search Strategy Document

*Does exposure to green or blue spaces impact dietary intake and food choices among adults – A systematic Review??*

# Search Strategy

| Database Name | Platform | Date Coverage | Date of Search | # of results |
| --- | --- | --- | --- | --- |
| 1. CINAHL Complete | EBSCO Host | 2010-Present | 14^th^ June 2021 | **1,252** |
| 2. GreenFILE | EBSCO Host | 2010-Present | 14^th^ June 2021 | **2,998** |
| 3. AMED | EBSCO Host | 2010-Present | 14^th^ June 2021 | **1** |
| 4. Medline | Full Text | 2010-Present | 14^th^ June 2021 | **6,122** |
| 5. PubMed | PubMed | 2010-Present | 14^th^ June 2021 | **524** |

**Total Records = 10,897**

**Duplicates = 716 (Deleted 716, Resolved 534)**

**Total Records after deduplication = 10,181**

| 6. Cochrane Library |  | 2010-Present | 14^th^ June 2021 | 18 |
| --- | --- | --- | --- | --- |

# Primary Search Strategy

**1. CINAHL Complete**

**Date of Search**: 14^th^ June 2021

**Number of results**: 1,252

| # | *Search string* | *# of results* |
| --- | --- | --- |
| S001 | Greenspace | 33,601 |
| S002 | Vegetation | 9,314 |
| S003 | Open space | 4,995 |
| S004 | Parklands | 39,985 |
| S005 | S001 OR S002 OR S003 OR S004 | 77,683 |
| S006 | Blue space | 50 |
| S007 | Surface waters | 9,362 |
| S008 | Water courses | 7,651 |
| S009 | S006 OR S007 OR S008 | 16,649 |
| S010 | Diet or diet* | 51,498 |
| S011 | Nutrition or Nutri* | 63,266 |
| S012 | Food choices | 8,398 |
| S013 | S010 OR S011 OR S012 | 84,561 |
| S014 | **S005 AND S009 AND S013** | **1,252** |

**2. Database name: GreenFILE**

**Date of Search:** 15^th^ June 2021

**Number of results**: 2,998

| **#** | **Search string** | **# of results** |
| --- | --- | --- |
| S001 | Greenspace | 72,892 |
| S002 | Vegetation | 67,551 |
| S003 | Open space | 16,752 |
| S004 | Parklands | 35,318 |
| S005 | S001 OR S002 OR S003 OR S004 | 161,900 |
| S006 | Blue space | 90 |
| S007 | Surface waters | 55,691 |
| S008 | Water courses | 33,636 |
| S009 | S006 OR S007 OR S008 | 80,522 |
| S010 | Diet or diet* | 12,840 |
| S011 | Nutrition or Nutri* | 29,946 |
| S012 | Food choices | 1,418 |
| S013 | S010 OR S011 OR S012 | 39,395 |
| S014 | S005 AND S009 AND S013 | **2,998** |

**3. Database name: AMED**

**Date of Search**: 14^th^ June 2021

**Number of results**: 1

| **#** | **Search string** | **# of results** |
| --- | --- | --- |
| S001 | Greenspace | 827 |
| S002 | Vegetation | 527 |
| S003 | Open space | 97 |
| S004 | Parklands | 1,561 |
| S005 | S001 OR S002 OR S003 OR S004 | 2,963 |
| S006 | Blue space | 1 |
| S007 | Surface waters | 216 |
| S008 | Water courses | 109 |
| S009 | S006 OR S007 OR S008 | 326 |
| S010 | Diet or diet* | 3,051 |
| S011 | Nutrition or Nutri* | 1,210 |
| S012 | Food choices | 64 |
| S013 | S010 OR S011 OR S012 | 3,754 |
| S014 | **S005 AND S009 AND S013** | **1** |

**4. Database name: Medline**

**Date of Search**: 15^th^ June 2021

**Number of results**: 6,122

| **#** | **Search string** | **# of results** |
| --- | --- | --- |
| S001 | Greenspace | 159,435 |
| S002 | Vegetation | 44,910 |
| S003 | Open space | 14,029 |
| S004 | Parklands | 164,515 |
| S005 | S001 OR S002 OR S003 OR S004 | 330,958 |
| S006 | Blue space | 293 |
| S007 | Surface waters | 36,893 |
| S008 | Water courses | 39,271 |
| S009 | S006 OR S007 OR S008 | 74,046 |
| S010 | Diet or diet* | 165,193 |
| S011 | Nutrition or Nutri* | 171,796 |
| S012 | Food choices | 17,145 |
| S013 | S010 OR S011 OR S012 | 259,626 |
| S014 | S005 AND S009 AND S013 | **6,122** |

**5. Database name: PubMed**

**Date of Search**: 14^th^ June 2021

**Number of results**: 524

| # | Search string | # of results |
| --- | --- | --- |
| S001 | Greenspace | 37,772 |
| S002 | Vegetation | 21, 600 |
| S003 | Open space | 262,235 |
| S004 | Parklands | 73,775 |
| S005 | S001 OR S002 OR S003 OR S004 | 375,915 |
| S006 | Blue space | 288 |
| S007 | Surface waters | 7,750 |
| S008 | Water courses | 19,912 |
| S009 | S006 OR S007 OR S008 | 27,766 |
| S010 | Diet or diet* | 96,398 |
| S011 | Nutrition or Nutri* | 107,178 |
| S012 | Food choices | 56,503 |
| S013 | S010 OR S011 OR S012 | 178,201 |
| **S014** | **S005 AND S009 AND S013** | **524** |

**6. Database name: Cochrane Library**

**Date of Search**: 10^th^ June 2021

**Number of results**: 18

| # | Search string | # of results |
| --- | --- | --- |
| S001 | Greenspace | 10,188 |
| S002 | Vegetation | 3,331 |
| S003 | Open space | 13,849 |
| S004 | Parklands | 11,410 |
| S005 | S001 OR S002 OR S003 OR S004 | 37,849 |
| S006 | Blue space | 60 |
| S007 | Surface waters | 1,250 |
| S008 | Water courses | 1,014 |
| S009 | S006 OR S007 OR S008 | 2,320 |
| S010 | Diet or diet* | 63,789 |
| S011 | Nutrition or Nutri* | 35,177 |
| S012 | Food choices | 4,697 |
| S013 | S010 OR S011 OR S012 | 80,527 |
| **S014** | **S005 AND S009 AND S013** | **18** |
