# Supplementary material for: The impact of exposure to green or bluespace on dietary intake and food choices among adults—A systematic literature review
Source: Food Sci Nutr. 2024 Nov 4;12(12):9913–27. doi: 10.1002/fsn3.4447 (PMC11666982; doi:10.1002/fsn3.4447)
Supplement: Supplementary file 1 — Data S1: [file FSN3-12-9913-s001.zip › S5 Critical Appraisal.zip!/S5 Critical Appraisal/Critical Appraisal Record 1.docx]

**JBI Critical Appraisal Checklist for Analytical Cross Sectional Studies**

**Reviewer: Claire Gilbourne	Date: 22^nd^ July 2021**

**Author: John W. M. Yuen et al.	Year: 2019	Record Number: 1**

**Title: *Influence of Urban Green Space and Facility Accessibility on Exercise and Healthy Diet in Hong Kong.***

| Question | Yes | No | Unclear | Not Applicable |
| --- | --- | --- | --- | --- |
| 1. Were the criteria for inclusion in the sample clearly defined? |  |  |  |  |
| 2. Were the study subjects and the setting described in detail? |  |  |  |  |
| 3. Was the exposure measured in a valid and reliable way? |  |  |  |  |
| 4. Were objective, standard criteria used for measurement of the condition? |  |  |  |  |
| 5. Were confounding factors identified? |  |  |  |  |
| 6. Were strategies to deal with confounding factors stated? |  |  |  |  |
| 7. Were the outcomes measured in a valid and reliable way? |  |  |  |  |
| 8. Was appropriate statistical analysis used? |  |  |  |  |

**Overall appraisal: 5/8 63%	Include****Exclude	Seek Further Info:**

**Comments (including reason for exclusion)**

1. ***Criteria for inclusion:*** *Criteria for inclusion and exclusion is described in sufficient detail*
2. ***Study subjects and setting:*** *Sample is described in sufficient detail and a clear description of the population from which the study participants were selected or recruited, including demographics, location, and time period is provided*
3. ***Measurement of Exposure:*** *Variables are clearly defined and accurately measured including exposure to green space.* *% of green space calculated using SPOT satellite image derived-derived dataset; addresses of participants were geocoded to the HK 1980 grid coordinates, and focal statistics of ArcGIS 10.6 (Environmental Systems Research Institute, Redlands, CA, USA) was applied to calculate % of green space within a 500m radius buffer around each participant’s residence.*
4. ***Criteria used for measurement of condition****: Not applicable, participants were selected based on convenience sampling methods and not included based on a specific diagnosis or definition.*
5. ***Confounding factors:*** *Its unclear if confounding factors were identified*
6. ***Strategies to deal with confounding factors:*** *Its unclear if strategies were incorporated to deal with confounding factors.*
7. ***Measurement of Outcomes:*** *Measurements of outcome for physical activity and diet were based on self-reported answers to structured questionnaire***,** *therefore the risk of over and under reporting is increased and objectivity is compromised. However assessment tools used were validated instruments including a validated 26 item Hong Kong version of WHOQOL-Brief used to assess four domains of QoL perception namely physical, psychological, social and environmental with 24 items, in addition to two individual items on general health and overall QoL. Chinese version of the International Physical Activity Questionnaire short form (IPAQ-C) was used to assess physical activity level of residents.*
8. ***Statistical Analysis:*** *Clear analytical techniques are described****.***
