# Supplementary material for: The impact of exposure to green or bluespace on dietary intake and food choices among adults—A systematic literature review
Source: Food Sci Nutr. 2024 Nov 4;12(12):9913–27. doi: 10.1002/fsn3.4447 (PMC11666982; doi:10.1002/fsn3.4447)
Supplement: Supplementary file 1 — Data S1: [file FSN3-12-9913-s001.zip › S5 Critical Appraisal.zip!/S5 Critical Appraisal/Critical Appraisal Record 2.docx]

**JBI Critical Appraisal Checklist for Analytical Cross Sectional Studies**

**Reviewer: Claire Gilbourne	Date: 25^th^ July 2021**

**Author: Paul Von Hippel & Rebecca Benson MPH	Year: 2014**

**Record Number: 2**

**Title: *Obesity and the Natural Environment Across US Counties***

| Question | Yes | No | Unclear | Not Applicable |
| --- | --- | --- | --- | --- |
| 1. Were the criteria for inclusion in the sample clearly defined? |  |  |  |  |
| 2. Were the study subjects and the setting described in detail? |  |  |  |  |
| 3. Was the exposure measured in a valid and reliable way? |  |  |  |  |
| 4. Were objective, standard criteria used for measurement of the condition? |  |  |  |  |
| 5. Were confounding factors identified? |  |  |  |  |
| 6. Were strategies to deal with confounding factors stated? |  |  |  |  |
| 7. Were the outcomes measured in a valid and reliable way? |  |  |  |  |
| 8. Was appropriate statistical analysis used? |  |  |  |  |

**Overall appraisal: 8/8 100%	Include****Exclude	Seek Further Info:**

**Comments (including reason for exclusion)**

1. ***Criteria for inclusion:*** *Criteria for inclusion and exclusion is described in sufficient detail, the inclusion/exclusion is specified, inclusion included residents aged 20 years+ and BMI >30*
2. ***Study subjects and setting:*** *Sample is described in sufficient detail (information collected from BRFSS, Behavioural Risk Factor Surveillance System measured in 2009 from 3100 counties of more than 350,000 adults)*
3. ***Measurement of Exposure:*** *Key regressors were measures of the natural environment, including weather variables (averaged across the years from 2004-2008). Snowfall data from Midwestern Regional Climate Center. Other weather variables from the National Oceanic and Atmospheric Administration, forest cover variable from the US Forest Service, other variables from the Economic Research Service of the US Department of Agriculture; topographic relief measured on a scale that runs from 1 for "flat plains" to 21 for "high mountains". Regression model accounted for spatial correlation and controlled for county demographics and the built environment. The measures of exposure are valid and reliable as the current measures of the natural environment are appropriate and can be repeated*
4. ***Criteria used for measurement of condition****: Yes, participants were included in the study based on a specified definition, ie BMI >30*
5. ***Confounding factors:*** *Yes confounding factors were identified and a longer list of control variables than have been used in past studies for obesity were controlled for. These included county demographics, built environment controls and two measures of air pollution.*
6. ***Strategies to deal with confounding factors:*** *Yes strategies to deal with confounding factors were dealt with in data analysis. The regression model was carefully specified with a large number of control variables and accounted for spatial correlation to ensure the model was less vulnerable.*
7. ***Measurement of Outcomes:*** *The outcome was an estimate of each countys 2009 prevalence of adult obesity, defined as the % of county residents aged 20 years and older whose BMI >30. These estimates were based on heights and weights collected by the Behavioral Risk Factor Surveillance System (BRFSS), a telephone survey of more than 350,000 US adults per year. As this information is self-reported, there is a risk of over and under reporting and objectivity is compromised. To correct for self-reported bias, this study used newly released bias corrected estimates of county obesity prevalence released separately for men and women.*
8. ***Statistical Analysis:*** *Clear analytical techniques are described****.*** *The study identified the variables* *included (dependent variable was an estimate of each countys 2009 prevalence of adult obesity; key regressors were measures of the natural environment, control variables included 15 measures of county demographics and 13 measures of county built environment).*
