# Supplementary material for: The impact of exposure to green or bluespace on dietary intake and food choices among adults—A systematic literature review
Source: Food Sci Nutr. 2024 Nov 4;12(12):9913–27. doi: 10.1002/fsn3.4447 (PMC11666982; doi:10.1002/fsn3.4447)
Supplement: Supplementary file 1 — Data S1: [file FSN3-12-9913-s001.zip › S5 Critical Appraisal.zip!/S5 Critical Appraisal/Critical Appraisal Record 3.docx]

**JBI Critical Appraisal Checklist for Analytical Cross Sectional Studies**

**Reviewer: Claire Gilbourne	Date: 26^th^ July 2021**

**Author: Yunwon Choi & Heeyeun Yoon	Year: 2020**

**Record Number: 3**

**Title: *Do the Walkability and Urban Leisure Amenities of Neighborhoods Affect the Body Mass Index of Individuals? Based on a Case Study in Seoul, South Korea***

| Question | Yes | No | Unclear | Not Applicable |
| --- | --- | --- | --- | --- |
| 1. Were the criteria for inclusion in the sample clearly defined? |  |  |  |  |
| 2. Were the study subjects and the setting described in detail? |  |  |  |  |
| 3. Was the exposure measured in a valid and reliable way? |  |  |  |  |
| 4. Were objective, standard criteria used for measurement of the condition? |  |  |  |  |
| 5. Were confounding factors identified? |  |  |  |  |
| 6. Were strategies to deal with confounding factors stated? |  |  |  |  |
| 7. Were the outcomes measured in a valid and reliable way? |  |  |  |  |
| 8. Was appropriate statistical analysis used? |  |  |  |  |

**Overall appraisal: 8/8 100%	Include****Exclude	Seek Further Info:**

**Comments (including reason for exclusion)**

1. ***Criteria for inclusion:*** *Criteria for inclusion and exclusion is described in sufficient detail, the inclusion/exclusion is specified, 577 residents were selected at random aged between 19 and 64 years of age and reside in Seoul from a national KNHNES (Korea National Health & Nutrition Examination Survey) collected in 2015.*
2. ***Study subjects and setting:*** *Sample is described in sufficient detail. Information collected from KNHNES study in 2015 was used to gain information on individual characteristics such as demographic information, BMI, SES, dietary pattern, physical activity and addresses. Built environment data was collected from a number of sources including National Geographic Information Institute (digital maps) and Statistical Geographic Information Service (GIS) provided by Statistics Korea. The sample size was 577 residents of Seoul aged between 19 and 64 years of age randomly selected from the 2015 survey out of 7380 individuals.*
3. ***Measurement of Exposure:*** *Three primary datasets were used in this study* *(KNHNES, SGIS and Korea National Geographic Information institute) to investigate the impact of neighborhood built environment (walkability and leisure amenities) on obesity (in interrelationship with socioeconomic status, SES)****.*** *The measures of exposure for the neighborhood built environment are valid and reliable as the current measures recorded and investigated are appropriate and can be repeated. Two measurements used for the construct SES, years of education and total household income are also valid and reliable.*
4. ***Criteria used for measurement of condition****: As genetic differences affect body fat patterns, different criterion are adopted for different ethnic groups, in Asia, BMI <18.5 is considered underweight, BMI of 18.5-23 represents an increasing but acceptable risk of being obese, 23-27.5 a heightened risk of being obese and 27.5 or higher a high risk of being obese.*
5. ***Confounding factors:*** *Yes two control constructs, physical activity and dietary pattern were controlled for in the analysis*
6. ***Strategies to deal with confounding factors:*** *Yes, the statistical analysis used, SEM* *differentiates between relationships as causal or association, direct or indirect and the second strength of the statistical analysis is its unbiased estimation of parameters. SEM considers measurement error, allowing the presence and correlation of error for each observed indicator in the model.*
7. ***Measurement of Outcomes:*** *The outcome variable was BMI, classified as per criterion established for ethnic groups. The information on the study sample was collected from a validated and reliable survey KNHNES 2015. This survey collects public health and nutritional status under Article 16 of the National Health Promotion Act. It includes health examination records, health interviews, nutrition records, personal information related to SES. While there is self-reporting by individuals in relation to information on health interviews, which can introduce self-reported bias, BMI status was collected from health examination records. Therefore, the outcome measurement on BMI is collected in a valid and reliable manner.*
8. ***Statistical Analysis:*** *Clear analytical techniques are described****.*** *Secondary data analysis using SEM (Structural Equation Modelling) to answer research questions via statistical analysis software, AMOS 22 (IBM, Chicago, IL, USA); (Quantitative Non randomized). Strength of SEM is its capacity to deduce complex relationships simultaneously among multiple endogenous (BMI) and exogenous latent constructs. Clear statistical analysis on walkability, urban leisure amenities and SES are presented.*
