# Supplementary material for: The impact of exposure to green or bluespace on dietary intake and food choices among adults—A systematic literature review
Source: Food Sci Nutr. 2024 Nov 4;12(12):9913–27. doi: 10.1002/fsn3.4447 (PMC11666982; doi:10.1002/fsn3.4447)
Supplement: Supplementary file 1 — Data S1: [file FSN3-12-9913-s001.zip › S5 Critical Appraisal.zip!/S5 Critical Appraisal/Critical Appraisal Record 4.docx]

**JBI Critical Appraisal Checklist for Analytical Cross Sectional Studies**

**Reviewer: Claire Gilbourne	Date: 25^th^ July 2021**

**Author:** **Akihiko Michimi & Michael C. Wimberly	Year: 2012**

**Record Number: 4**

**Title: *Natural environments, obesity, and physical activity in nonmetropolitan areas of the United States***

| Question | Yes | No | Unclear | Not Applicable |
| --- | --- | --- | --- | --- |
| 1. Were the criteria for inclusion in the sample clearly defined? |  |  |  |  |
| 2. Were the study subjects and the setting described in detail? |  |  |  |  |
| 3. Was the exposure measured in a valid and reliable way? |  |  |  |  |
| 4. Were objective, standard criteria used for measurement of the condition? |  |  |  |  |
| 5. Were confounding factors identified? |  |  |  |  |
| 6. Were strategies to deal with confounding factors stated? |  |  |  |  |
| 7. Were the outcomes measured in a valid and reliable way? |  |  |  |  |
| 8. Was appropriate statistical analysis used? |  |  |  |  |

**Overall appraisal: 5/8 63%	Include****Exclude	Seek Further Info:**

**Comments (including reason for exclusion)**

1. ***Criteria for inclusion:*** *Criteria for inclusion and exclusion is described in sufficient detail, the inclusion/exclusion is specified, inclusion included residents residing in non-metropolitan counties as defined by the Office of Management and Budget aged 18 years+ and non-institutionalized*
2. ***Study subjects and setting:*** *Sample is described in sufficient detail (information collected from BRFSS, Behavioural Risk Factor Surveillance System measured in 2000-2006, non-institutionalized residents residing in non-metropolitan counties as defined by the Office of Management and Budget were selected, number of non-metropolitan counties included in the analysis was 2,013. BRFSS respondents provided information about their height and weight from which their BMI was computed, for physical activity respondents were classified into those who reported engaging in physical activity or exercise during the past 30 days and those who did not). The sample size was 457,820 for obesity and 473,296 for physical activity.*
3. ***Measurement of Exposure:*** *Two environmental indices of outdoor activity potential (OAP) were examined to test hypothesis, a recreational opportunity index and a natural amenities index. The recreational opportunity index was based on measurements of outdoor recreation facilities. Data was obtained from the 1997 National Outdoor Recreation Supply Information System (NORSIS) by the USDA Forest Service Southern Research Station. 24 variables were selected from NORSIS. The natural amenities index was based on measurements of physiography and cover, climate, and tourism employment, this index was composed of 8 variables. The measures of exposure for OAP are valid and reliable as the current measure is appropriate and can be repeated.*
4. ***Criteria used for measurement of condition****: Yes, participants were included in the study based on a specified definition, i.e. non-institutionalized residents residing in non-metropolitan counties as defined by the Office of Management and Budget.*
5. ***Confounding factors:* Individual level characteristics were identified**.
6. ***Strategies to deal with confounding factors:*** *Covariates in the regression model included age, sex, race/ethnicity, and income to control for individual level characteristics in the analysis*
7. ***Measurement of Outcomes:*** *The outcome variables were obesity and physical activity with individual level data obtained from BRFSS 200-2006. These estimates were based on heights and weights collected by the Behavioral Risk Factor Surveillance System (BRFSS), a telephone survey that provides state-specific data on health risk behavior and other information related to chronic disease. Self-reported data on height and weight is often biased and objectivity is compromised. Physical activity was examined in one broad metric and as such if measurements of physical activity were repeated, different results maybe obtained. Therefore, the outcomes are not measured in a reliable way.*
8. ***Statistical Analysis:*** *Clear analytical techniques are described****.*** *The study identified the outcome variables as physical activity and obesity. Covariates* *included age, sex, race/ethnicity, education and income. Odds ratios (OR) and 95% confidence intervals (CI) were reported for each model. County level intra-correlation was accounted for using SAS GENMOD procedure.*
