# Supplementary material for: The impact of exposure to green or bluespace on dietary intake and food choices among adults—A systematic literature review
Source: Food Sci Nutr. 2024 Nov 4;12(12):9913–27. doi: 10.1002/fsn3.4447 (PMC11666982; doi:10.1002/fsn3.4447)
Supplement: Supplementary file 1 — Data S1: [file FSN3-12-9913-s001.zip › S5 Critical Appraisal.zip!/S5 Critical Appraisal/Second Reviewer appraisal/JBI Critical Appraisal Checklist Hippel.docx]

**JBI Critical Appraisal Checklist for Analytical Cross Sectional Studies**

**Reviewer: Alan Scarry	Date: 22^nd^ July 2021**

**Author: Hippel et al.	Year: 2014	Record Number: 2**

**Title: *Influence of Urban Green Space and Facility Accessibility on Exercise and Healthy Diet in Hong Kong.***

| Question | Yes | No | Unclear | Not Applicable |
| --- | --- | --- | --- | --- |
| 1. Were the criteria for inclusion in the sample clearly defined? |  |  |  |  |
| 2. Were the study subjects and the setting described in detail? |  |  |  |  |
| 3. Was the exposure measured in a valid and reliable way? |  |  |  |  |
| 4. Were objective, standard criteria used for measurement of the condition? |  |  |  |  |
| 5. Were confounding factors identified? |  |  |  |  |
| 6. Were strategies to deal with confounding factors stated? |  |  |  |  |
| 7. Were the outcomes measured in a valid and reliable way? |  |  |  |  |
| 8. Was appropriate statistical analysis used? |  |  |  |  |

**Overall appraisal:	Include****Exclude	Seek Further Info:**

**Comments (including reason for exclusion)**

1. ***Criteria for inclusion:*** *No criteria for inclusion, regression model used and physical activity and diet were used as mediators.*
2. ***Study subjects and setting:*** *Yes detail was given about the study subject matter*
3. ***Measurement of Exposure:*** *Yes this was measured using the dependent variable as an estimate of each county’s 2009 prevalence of adult obesity, defined as the percentage of county residents aged 20 years and older whose BMI exceeded 30.*
4. ***Criteria used for measurement of condition****: Yes as the same set of criteria was used in all 3100 countries.*
5. ***Confounding factors:*** *Under reporting of weight and over reporting of height.*
6. ***Strategies to deal with confounding factors:*** *Yes the* newly released estimates of county obesity prevalence, which correct self-report bias.
7. ***Measurement of Outcomes*** *Linear regression model were used*
8. ***Statistical Analysis:*** *Clear statistical analysis was used.*
