# Supplementary material for: The impact of exposure to green or bluespace on dietary intake and food choices among adults—A systematic literature review
Source: Food Sci Nutr. 2024 Nov 4;12(12):9913–27. doi: 10.1002/fsn3.4447 (PMC11666982; doi:10.1002/fsn3.4447)
Supplement: Supplementary file 1 — Data S1: [file FSN3-12-9913-s001.zip › S5 Critical Appraisal.zip!/S5 Critical Appraisal/Second Reviewer appraisal/JBI Critical Appraisal Checklist Yuen et al.docx]

**JBI Critical Appraisal Checklist for Analytical Cross Sectional Studies**

**Reviewer: Alan Scarry	Date: 22^nd^ July 2021**

**Author: Yuen et al.	Year: 2019	Record Number: 1**

**Title: *Influence of Urban Green Space and Facility Accessibility on Exercise and Healthy Diet in Hong Kong.***

| Question | Yes | No | Unclear | Not Applicable |
| --- | --- | --- | --- | --- |
| 1. Were the criteria for inclusion in the sample clearly defined? |  |  |  |  |
| 2. Were the study subjects and the setting described in detail? |  |  |  |  |
| 3. Was the exposure measured in a valid and reliable way? |  |  |  |  |
| 4. Were objective, standard criteria used for measurement of the condition? |  |  |  |  |
| 5. Were confounding factors identified? |  |  |  |  |
| 6. Were strategies to deal with confounding factors stated? |  |  |  |  |
| 7. Were the outcomes measured in a valid and reliable way? |  |  |  |  |
| 8. Was appropriate statistical analysis used? |  |  |  |  |

**Overall appraisal:	Include****Exclude	Seek Further Info:**

**Comments (including reason for exclusion)**

1. ***Criteria for inclusion:*** *This paper meets the inclusion criteria.*
2. ***Study subjects and setting:*** *adult residents of any gender aged at least 20 years and had been living in any of the nine districts for more than one year were the target population.*
3. ***Measurement of Exposure:*** *The exposure: green space was calculated based on a spatial buffer with a 500 m radius from participants’ geocoded addresses using a SPOT (‘Satellite Pour l’Observation de la Terre’ in French)* *satellite image-derived vegetation dataset. Parks, promenade and sports facilities were examples of open spaces.*

4. ***Criteria used for measurement of condition****: Not applicable, Participants were selected using convenience sampling.*
5. ***Confounding factors:*** *It is unclear from this paper if there were confounding factors*
6. ***Strategies to deal with confounding factors:*** *It is unclear from this paper if there were confounding factors.*
7. ***Measurement of Outcomes:*** *Measurement was carried out in as valid and reliable way as possible give that this type of research carries risk, methods were put in place to mitigate the risk and this was done comprehensively throughout the study.*
8. ***Statistical Analysis:*** *Clear statistical analysis was used.*
